# Improving Hand hygiene Compliance at Selected Health Facilities in Uganda Using the WHO Multimodal Strategy

**DOI:** 10.1017/ash.2025.337

**Published:** 2025-09-24

**Authors:** Shillah Nakato, Martin Esagala

**Affiliations:** 1 Infectious Diseases Institute, Makerere University; 2 IDI

## Abstract

**Background:** Uganda has a high prevalence of healthcare associated infections (HCAIs) with 28% often linked to inadequate hand hygiene practices among health workers. Hand hygiene is one of the most important measures in reducing the transmission of nosocomial infections. Implementing a world health organization (WHO) multimodal hand hygiene improvement strategy has shown influence on health workers’ behaviors, knowledge and practices. We aimed at evaluating hand hygiene compliance among health workers before and after implementation of the WHO multimodal improvement strategy at select health facilities(HF) in Uganda. **Method:** 27 health facilities were randomly selected from two regions in Uganda to implement the WHO multimodal hand hygiene improvement strategy over a period of 4 weeks. Before the interventions, healthcare worker’ (HCW) compliance with hand hygiene during routine patient care was directly observed using the WHO hand hygiene observation tool. Interventions included; weekly onsite mentorships focusing on Training and education, provision of locally produced alcohol-based hand rubs (ABHR), soap, and placement of reminders such as posters at point of care areas to emphasize the importance of hand hygiene. HCWs from different facility departments were designated to champion hand hygiene. We recorded and distributed hand hygiene promotional videos to the health facilities to reinforce key messages consistently. After the interventions, follow up observations were conducted and data was analyzed using SPSS 20. **Results:** A total of 156 health workers were observed at baseline and 151 at follow on. 1,205 hand hygiene opportunities were recorded at baseline and 1,369 at follow on. 454 actions were observed at baseline and 845 after. Healthcare worker hand hygiene compliance improved from 32.6% (SD=23) to 60.7% (SD=23; p=0.0083) after the intervention. The increase in compliance to hand hygiene was different across all professional categories with significant improvement among Lab technicians (72% Versus 35%). Compliance among students remained low at 36% versus 38% post intervention. 76% of the observed health workers preferred use of locally produced ABHR while 22% used water and soap. **Conclusion:** The improvement in hand hygiene compliance among health workers following short term interventions using the WHO multimodal improvement strategy shows potential effectiveness. This underscores the importance of prolonged commitment from hospitals in adopting and reinforcing this strategy for long-term improvements in hand hygiene practices among health workers.

